# Effects of Diluent pH on Enrichment and Performance of Dairy Goat X/Y Sperm

**DOI:** 10.3389/fcell.2021.747722

**Published:** 2021-10-01

**Authors:** Qifu He, Shenghui Wu, Ming Huang, Ying Wang, Kang Zhang, Jian Kang, Yong Zhang, Fusheng Quan

**Affiliations:** ^1^Key Laboratory of Animal Biotechnology, Ministry of Agriculture, Yangling, China; ^2^College of Veterinary Medicine, Northwest A&F University, Xianyang, China; ^3^Key Laboratory of Animal Biotechnology, Northwest A&F University, Xianyang, China

**Keywords:** sperm, gender control, diluent pH, IVF, dairy goat

## Abstract

In this paper, on the basis of the differences in the hydrogen ion concentration (pH) of the diluent dairy goat semen on X/Y sperm motility, an X/Y sperm enrichment study was conducted to establish a simple and effective method for gender control in dairy goats. Dairy goat semen was diluted using different pH dilutions and was incubated. Then, the X/Y sperm ratio in the isolated upper sperm was determined using the double TaqMan qPCR method. The internal pH change pattern of sperm cells at different pH dilutions was measured using BCECF-AM probe, and the functional parameters of the isolated sperm were tested with the corresponding kit. Next, an *in vitro* fertilization test was conducted using isolated spermatozoa and oocytes to determine their fertilization rates, the percentages of female embryos, and the expression of genes related to developing potentially fertilized embryos. Results showed that the percentages of the X sperm cells in the upper sperm layer were 67.24% ± 2.61% at sperm dilution pH of 6.2 and 30.45% ± 1.03% at sperm dilution pH of 7.4, which was significantly different from 52.35% ± 1.72% of the control group (pH 6.8) (*P* < 0.01). Results also showed that there is a relationship between the external pHo and internal pHi of sperm cells. Furthermore, the percentages of female embryos after the *in vitro* fertilization of the isolated upper sperm with mature oocytes at pH 6.2 and 7.4 were 66.67% ± 0.05 and 29.73% ± 0.04%, respectively, compared with 48.57% ± 0.02% in the control group (pH 6.8). Highly significant differences occurred between groups (*P* < 0.01). Additionally, no significant difference was observed during the expression of genes related to embryonic development between the blastocysts formed from sperm isolated by changing the pH of the diluent and the control sperm (*P* > 0.05). Therefore, this study successfully established a simple and effective method for enriched X/Y sperms from dairy goats, which is important for regulating the desired sex progeny during dairy goat breeding and for guiding dairy goat production.

## Introduction

Gender control technology is a breeding technology that artificially intervenes and makes female animals produce offspring of the desired sex according to the breeder’s wishes, thereby making it a hot topic in animal breeding and livestock production research (Hansen, 2014; Xie et al., 2020). By controlling the sex of a herd, the proportion of expected sex offspring can be increased. Therefore, female livestock can give full play to reproduction and lactation performance, and male livestock can give full play to meat or service performance, therefore improving the efficiency of livestock breeding (Johnson, 2000). Furthermore, studying the subtle differences that exist between X and Y sperms, and using their differences to separate X and Y sperms, thereby fertilizing them with eggs is the most effective way to intervene and select the sex of offspring (Rahman and Pang, 2019). Researchers have also separated X and Y sperms by electrophoresis based on the differences in cell surface charge contents (Ishijima et al., 1992). Specific ligands have also been used to bind sperm-specific receptors, such as Y sperm SRY proteins (Soleymani et al., 2019), HY antigen (Yadav et al., 2017), and TLR7/8 receptor (Umehara et al., 2020). The most effective and frequently used method for sperm separation is flow cytometry sorting, which takes advantage of the different DNA contents of X and Y sperms. This method leads to different quantities of binding to nucleic acid dyes (Ortega-Ferrusola et al., 2017). However, the viability and fertilization capacity of dairy goat sperm isolated using the above methods are significantly reduced. Additionally, DNA quality and cell membrane integrity are compromised, which induces apoptosis. Flow cytometry sorting equipment is expensive and requires specialized personnel to operate, thereby making its wide use difficult (Said and Land, 2011; Soares et al., 2019).

Mammalian *in vitro* fertilization has become an important and routine biotechnology for animal reproduction (Jochems et al., 2021; Redel et al., 2021; Seekford et al., 2021), which requires high-quality oocytes, and is closely related to sperm quality (Rowlison et al., 2021; Shah and Andrabi, 2021). The pH environment to which semen is exposed can affect the motility and metabolic level of spermatozoa, with metabolism and motility being hindered and motility being diminished in a weakly acidic environment. Nevertheless, these characteristics are enhanced in a weakly alkaline environment (Zhou et al., 2015; Wurlina et al., 2020). Sperm swims forward (upward) in their natural state, therefore aiding the separation of X/Y sperms by changing the incubation conditions (Shi et al., 2021). Furthermore, mammalian X and Y spermatozoa differ in their motility activity under different pH dilutions. Thus, researchers have incubated rabbit sperms under different pH dilutions for fertilization, and the sex ratio of the offspring was significantly altered (Muehleis and Long, 1976). Gopher sperm also decreased the proportion of male offspring when the pH of the reproductive tract was increased, with a significantly negative correlation (Pratt et al., 1987). Additionally, the X/Y sperm ratio in the upper sperm was altered when human sperm was incubated under different pH dilutions (Oyeyipo et al., 2017; You et al., 2017). Therefore, as dairy goats are important milk-producing livestock (Menchaca et al., 2020), it is important to change the sex of the offspring by artificially breeding dairy goats. The most direct and simple method is through the effective separation of X/Y sperm in dairy goats. It is also significant to screen the dilutions of pH that can effectively enrich the X/Y sperm to achieve the purpose of artificially controlling the sex of the offspring while ensuring normal fertilization rates.

## Materials and Methods

### Materials

The dairy goat semen was collected from purebred Saanen dairy goats raised at the China Cloning Animal Base in Yangling Demonstration Zone, Shaanxi Province. A pseudovaginal method was used to collect semen three times from 10 healthy rams aged 1.5–2 years (volume ≥ 1 mL, viability ≥ 0.7), with an interval of 24 h between each collection.

Subsequently, ovaries from 1- to 2-year-old goats were provided by a local abattoir in Baoji (100 km) and returned to the laboratory within 2 h after collection (incubator setting 25°C ± 2°C), after which ovarian tissues were dissociated according to the established laboratory standards of Northwest A&F University.

### Reagents

All chemicals and reagents were purchased from Sigma-Aldrich (St. Louis, MO, United States), unless otherwise indicated.

DNA Marker 100/2000 (3422A; TaKaRa), *Escherichia coli* DH5α Competent cells (9057; TaKaRa), Plasmid extraction kit (PLN350; Sigma-Aldrich), T-Vector pMD^TM^ 19 (3271; TaKaRa).

### Experimental Design

This section briefly outlines the experiments undertaken in this study. Experiment 1 established a double TaqMan qPCR method and used it to investigate whether the upper X/Y sperm ratio changes when sperm is incubated under different pH dilutions. Experiment 2 investigated the effect of different pH dilutions on the internal pH of dairy goat spermatozoa. Experiment 3 examined whether the performance and quality of isolated upper layer spermatozoa are altered, including sperm acrosome integrity, sperm plasma membrane integrity, sperm DNA fragmentation, sperm adenosine triphosphate (ATP) content, sperm mitochondrial activity, sperm reactive oxygen species (ROS) content, total sperm motility, and forward motility. However, experiment 4 investigated the *in vitro* fertilization rate of isolated upper sperm, embryo sex ratio, and mRNA expression of genes based on the blastocyst’s development potential to evaluate blastocyst quality.

Experimental group allocations were as follows. The base formulation of the sperm diluent used in the experiment included glucose (55.51 mmol/L), fructose (55.51 mmol/L), lactose (29.21 mmol/L), ethylene diamine tetraacetic acid (EDTA) (20.82 mmol/L), penicillin (100 U/mL), and streptomycin (100 U/mL). However, the osmolality of the diluent was adjusted to 330 mOsmol/kg. The diluent was also prepared using a citric acid (10.93 mmol/L)–sodium citrate (112.37 mmol/L) buffer pair. Control group: diluent pH was 6.8 (pH of semen in its natural state). Experimental group: diluent pH was adjusted to 5.8, 6.2, 6.6, 7.0, 7.4, and 7.8 using NaOH and HCL.

### Determining Upper X/Y Sperm Ratio After Incubation Under Different Hydrogen Ion Concentration Dilutions

Ten fresh semen samples (six replicates for each sample) were diluted to 600 × 10^4^/mL using the pH 6.8 diluents, and it was centrifuged at 1,000 × *g* for 5 min. The supernatant was discarded and added slowly with 10 mL different pH diluent. Then, the tubes were tilted to 45° and incubation at 37°C with 5% CO_2_. Finally, 10 μL upper sperm was taken at 10 min intervals, and genomic DNA was extracted using a DNAiso Reagent kit (9770A; TaKaRa).

To calculate the X/Y sperm ratios in isolated upper sperm and analyze the X and Y sperm bioinformatics of dairy goats, primers were designed by selecting the F9 gene specific to the X chromosome and the ZFY gene specific to the Y chromosome (Table 1). The genomic DNA of dairy goat sperm was used as a template for PCR amplification. The PCR product was ligated to T-Vector pMD^TM^ 19 and transferred to *E. coli* DH5α competent cells by the heat shock method to prepare a positive standard. By optimizing the PCR reaction system and reaction conditions, we established a standard curve and verified the specificity, sensitivity, and stability of the method. Subsequently, the double TaqMan qPCR (Thermal Cycler CFX96 Real-Time System, United States) method was employed to calculate the X/Y sperm ratio.

**TABLE 1:** Primer information for PCR.

**Target gene**	**Primer sequence (5′–3′)**	**Tm (°C)**	**Fragment size (bp)**	**Reference sequence**
F9	F: GCCACGTGTCTTCGATCCAC R:CCCCACTGTCTCCTTGGCAT Probe:HEX -TTCTGTGCCGGCTACCATGAGGGAGGT- TAMRA	60	100	XM_004022305.4 (1,168–1,248)
ZFY	F: CCACGTCAAGCGACCCAT R:AGAGCCACCTTTCGTCTTCG Probe:FAM -AACGCCTTCATTGTGTGGTCTCGTGA- BHQ	60	66	MF741782.1 (534–599)
OCT4	F: CGCCCTATGACTTGTGTGGA R:GGCTGAGGGGTCTCCAGG	59	91	NM_001285569.1 (194–284)
NANOG	F: ACCCGGAGATCTTCACCTTTC R:CAAGCTGGGTCCACACTCAT	59	129	JQ801747.1 (1–129)
PGRMC1	F: CGCTGGCTAGTCTTGGTCAG R:TGATCGCTGGAGCAAAGGTT	60	113	XM_018044217.1 (192–304)
COX2	F: CGTCCAGGCCTATTCTACGG R:GGGACTAGCTCGAGAACGAT	58	83	AB736135.1 (562–644)
PLAC8	F: AGGACCCTCTACAGGACTCG R:AAAGTGCGATTGGCTCTCCT	59	134	XM_005681847.3 (347–480)
TP53	F: GAAGAGGGTGCTAAGAGCGG R:GGCGCTTCATTCGGACATTC	60	230	XM_018064593.1 (8–237)
BAX	F: CAGCAAACTGGTGCTCAAGG R:AGCCGCTTTGGAAGGAAGT	59	93	JN036558.1 (70–162)
SOD2	F: GCTGGAAGCCATCAAACGTGC R:AGCAGGGGGATAAGACCTGT	60	189	XM_018053428.1 (478–666)
GSTM3	F: TACTGGGATATTCGCGGGCT R:TTCCCCGCACGTGTATCTTT	60	93	XM_005677937.3 (130–222)
*GAPDH*	F: TCCTGCCCGTTCGACAGA R:TTGATGACGAGCTTCCCGTT	60	265	XM_005680968.3 (44–308)
*Aml-X*	F: CAGTAGCTCCAGCTCCAGCT R:GTGCATCCCTTCATTGGC	58	300	AF 215887.1 (102–401)
*SRY-Y*	F: ATGAATAGAACGGTGCAATCG R:GAAGAGGTTTTCCCAAAGGC	58	116	MF 741782.1 (346–461)

### Detecting the Effect of Diluent Hydrogen Ion Concentration on the Intracellular Hydrogen Ion Concentration of Spermatozoa

Determination of pH changes inside and outside sperm cells with reference to the experimental procedure Achikanu et al. (2018) was conducted. The semen was diluted to 600 × 10^4^/mL with the pH 6.8 diluent. Then, 1 mL of the diluted semen was centrifuged at 1,000 × *g* for 5 min. Subsequently, the supernatant was discarded, after which we added and resuspended 1 mL of different pH dilutions into the setup. After 1 min, 100 μL of sperm was taken at 1 min intervals, and then, we added the cell membrane permeabilizer 12% Triton X-100 (final concentration, 0.12%) (9002-93-1, Sigma-Aldrich) to permeabilize the cell membrane. After centrifugation at 1,000 × *g* for 5 min, the supernatant was discarded, and the sample was extracted using a PBS FLUO star ELISA (BMG Labtech Offenburg, Germany) at 2′,7′-Bis(2-carboxyethyl)-5(6)- carboxyfluorescein, acetoxymethyl ester (BCECF, AM)-loaded cells using excitation wavelength 490/440 nm to determine the intracellular emission wavelength 530 nm fluorescence ratio in sperm cells (six replicates for each sample), using non-linear regression in Graphpad Prism 8.0 (curve fit) was conducted to establish the relationship between pH and the internal emission wavelength 530 nm fluorescence ratio of sperm cells at excitation wavelength 490/440. Subsequently, the sperm cells were incubated in different pH dilutions for 300 s, washed with PBS, and centrifuged at 1,000 × *g* for 5 min, after which the supernatant was discarded. The internal emission fluorescence 530 nm ratio of sperm cells at excitation wavelength 490/440 nm was measured by enzyme marker, and the internal pH of sperm was calculated by the above fitted curve (six replicates for each sample). Finally, the internal pH of sperm was calculated using GraphPad Prism 8.0, and linear regression was used to establish the equation for the internal pH when the sperm cells were at different pH values.

### Determining Performance and Quality of Upper Spermatozoa After Incubation With Different Hydrogen Ion Concentration Dilutions

Semen samples were diluted using pH 6.8 diluents to 600 × 10^4^/mL, and then, we measured 10 mL of semen and centrifuged it at 1,000 × *g* for 5 min. We subsequently discarded the supernatant and slowly added 10 mL of the diluent at different pH levels. Next, we placed the glass tube in a 37°C, 5% CO_2_ incubator at an inclination of 45° and measured 1 mL of the upper sperm after incubating for 40 min. Following the instructions of the sperm quality test kit, we determined the functional parameters of sperm samples in turns (six replicates for each sample). The intactness of the sperm acrosome was detected using peptide nucleic acid (PNA) fluorescence staining (031; BRED), the intactness of the sperm plasma membrane was detected using a LIVE/DEAD Sperm Viability Kit (L7011; Thermo Fisher), the degree of sperm DNA fragmentation was analyzed using Sperm Chromatin Structure Analysis (SCSA) (20160051; Cellpro), and sperm ATP content was determined using an ATP Assay Kit (EATP-100; BioAssay Systems). Furthermore, determination of sperm mitochondrial activity was conducted using JC-1 fluorescent probes (C2005; Beyotime), determination of sperm ROS content was conducted using ROS detection kits (S0033S; Beyotime), and determination of total motile sperm percentage and forward motile sperm ratios was conducted using a sperm analyzer (SJ-TMDI608; MaiLang). Detailed steps of the above kit operation are shown in Supplementary Material 1.

### Detecting the Sex Ratio of Embryos and mRNA Expression of Genes Related to Blastocyst Development After Enrichment

The returned ovaries were washed three times with prewarmed (30°C) sterilized saline, after which excess fat and connective tissues were cut off using scissors. The prepared tissue was subsequently placed in a flat dish containing an egg collection solution (TCM199 + 1% FBS + 100 mg/mL sodium heparin + 100 IU/mL penicillin + 100 IU/mL streptomycin). Next, 2 ∼ 6 mm follicles were cut on the surface of the ovaries using a sterile scalpel. The follicles were then released from the follicular fluid and the oocyte complex of the oocyte mound (COCs). COCs with three or more layers of oocytes, densely packed, and comprising homogeneously black oocyte cytoplasms were selected under the microscope. In further steps, *in vitro* maturation of oocytes in basic culture medium (M199 medium + 1 μg/mL E2 + 10 μg/mL FSH + 10 μg/mL LH + 20% EGS) was conducted for 24–27 h (38.5°C, 5% CO_2_, and saturated humidity) to release the first polar bodies as maturity indicators.

The sperm isolated after incubation under different pH conditions was centrifuged at 1,000 × *g* for 5 min at room temperature, and then, the supernatant was discarded and resuspended in a dilution solution at pH 6.8. Subsequently, sperm concentration was adjusted to 100 × 10^4^/mL. In the next step, at 1:1 by volume, 50 μL of the equilibration solution (5 mg/mL BSA + 0.5 mg/mL caffeine + 0.3 mg/mL glutathione + 20 μg/mL heparin) was added to make fertilization sperm microdrops. This step was conducted in an incubator (38.5°C, 5% CO_2_, and saturated humidity) for 60 min while the control group was being set up (dilution pH 6.8). Every 30 mature oocytes were then transferred into one fertilized microdroplet and coincubated for 20 h. Furthermore, the sperm and other oocytes surrounding the oocytes were removed and transferred to the embryo culture medium (50 mL mSOF + 6 mg/mL BSA) for further incubation. Finally, the *in vitro* fertilization rate of oocytes was observed and recorded. The release of the second polar body and the formation of a zygote between the female pronucleus and the male pronucleus was used as a criterion to determine whether the oocytes were fertilized or not.

Next, embryos were collected (single embryos) at the blastocyst stage of development (6–8 days) and transferred to PBS containing 5 mg/mL protease (Sigma, Madrid, Spain) for 1 min to remove the zona pellucida. Then, 8 μL of 100 μg/mL proteinase K (P8044-1G; Sigma) was added at 37°C overnight to thoroughly lyse the embryonic cells. Subsequently, 3 μL of the lysis product was taken for duplex PCR (Phua et al., 2003) amplification to detect the sex of each embryo (three replicates for each sample), the primers are shown in Table 1.

To study the relative mRNA expression of genes related to blastocyst developmental potentials, after sex verification of embryos from the same pH group, every 5 blastocyst cleavage product from the samples of the same sex (2 μL) was then pooled and added to remove genomic DNA enzymes (RR047A; TaKaRa). Reverse transcription was used to obtain cDNA using a kit (RR047A; TaKaRa). The genes tested included OCT4, NANOG, PGRMC1, COX2, PLAC8, TP53, BAX, GSTM3, and SOD2 (three replicates for each sample). The primers are shown in Table 1. Next, the comparative *C*_T_ method was used to calculate the relative quantity of the target gene mRNA, normalized to *GAPDH* and relative to the calibrator, and was expressed as the fold change = 2^–ΔΔCt^ (Bermejo-Alvarez et al., 2008). The following conditions were applied to the qPCR experiments: polymerase activation and template denaturation for 30 s at 95°C, followed by 40 cycles of denaturation for 5 s at 95°C, annealing for 30 s at 60°C, and then extensions for 30 s at 72°C.

### Statistical Analysis

Statistical analysis was performed using GraphPad Prism 8.0 software (GraphPad Software Inc., San Diego, CA, United States). Data were expressed as the mean ± standard deviation of at least three independent experiments. The differences in variables between groups were evaluated by Student’s *t*-test (two-tailed). Multiple comparisons between groups were performed using one-way analysis of variance and an LSD test. *P*-values < 0.05 were considered statistically significant.

## Results

### X/Y Sperm Ratios of the Upper Layer After Incubating With Different Hydrogen Ion Concentration Dilutions

#### Establishing TaqMan qPCR Method for Calculating the X/Y Sperm Ratio

**FIGURE 1:**
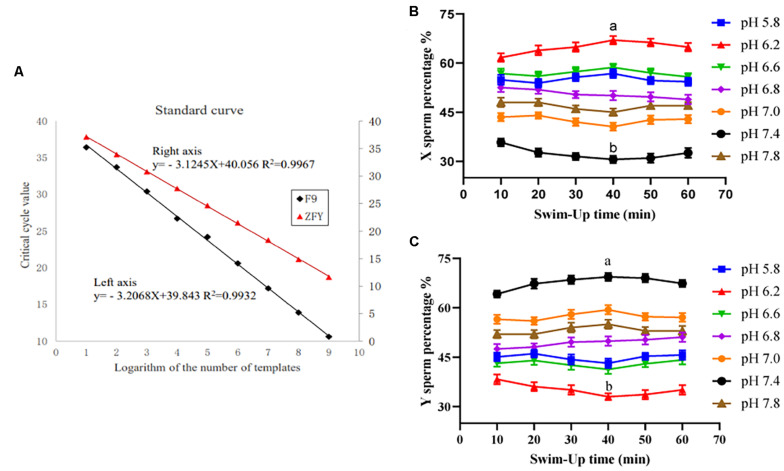
Double TaqMan qPCR method standard curve and the upper sperm proportion after incubation. **(A)** Double TaqMan qPCR method standard curve. **(B)** The proportion of the upper X sperm present in different pH dilution solutions for different times. **(C)** The proportion of the upper Y sperm present in different pH dilution solutions for different times. “a,b” mean significantly different from each other (*P* < 0.05).

The minimum detection amounts for F9 and ZFY genes were also placed at 47 and 51 copies/μL, respectively. The standard curves were *y* = −3.2068*x* + 39.843, *R*^2^ = 0.9932, and *y* = −3.1245*x* + 40.056, *R*^2^ = 0.9967, respectively. Results are shown in Figure 1A.

#### Analyzing the Ratio of X/Y Sperm Samples in the Upper Layer After Incubating With Different Hydrogen Ion Concentration Diluents

The X/Y sperm ratio was calculated based on the *C*t value of real-time fluorescence PCR amplification and the standard curve. When the sperm diluent pH was 6.2 and incubated for 40 min, the percentage of X sperm cells in the upper sperm was the highest (67.24% ± 2.61%), and was significantly higher than that of the control group (52.35% ± 1.72%). However, when the diluent pH was 7.4 and cultivated for 40 min, the percentage of the Y sperm in the upper sperm was the highest (69.53% ± 3.04%), and was significantly higher than that of the control group (48.12% ± 2.18%). Results are shown in Figures 1B,C.

### Analysis of the Relationship Between the Diluent Hydrogen Ion Concentration and Intracellular Hydrogen Ion Concentration of Spermatozoa

**FIGURE 2:**
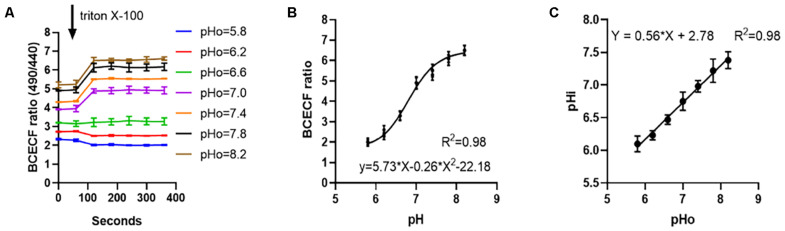
The relationship between internal pH changes of sperm in different pH diluents. **(A)** Measure the internal excitation wavelength 490/440 nm ratio of sperm changes with time at different pH. **(B)** Fitting curve of the ratio of pH to sperm internal excitation wavelength 490/440 nm. **(C)** Linear relationship between the pH of the diluent and the internal pH of the sperm.

After the sperm cells were incubated at different pH dilutions and the cell membrane permeabilizer was added, the ratio of fluorescence intensity of 530 nm at excitation wavelengths of 490 and 440 nm was measured, and the ratio changes with time are shown in Figure 2A. The relationship between this value and the internal pH of sperm cells was *y* = 5.73*x* − 0.26*x*^2^ − 22.18, *R*^2^ = 0.98 (Figure 2B). Moreover, the relationship with internal pH when the sperm cells were subjected to different pH dilutions was *y* = 0.56*x* + 2.78, *R*^2^ = 0.98. Results were shown in Figure 2C.

### Effects of Hydrogen Ion Concentration Diluent on the Integrity of Sperm Acrosome and the Plasma Membrane

**FIGURE 3:**
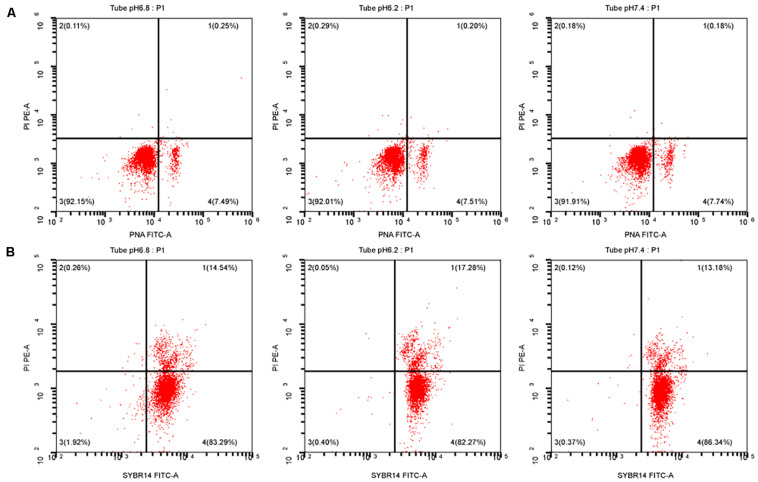
Test for the performance of the upper enriched sperm. **(A)** Sperm acrosome integrity test result. **(B)** Sperm plasma membrane integrity test result. PI, a fluorescent dye that binds to the DNA of apoptotic cells; PNA-FITC, green fluorescein-labeled peanut agglutinin, which binds to β-D-galactosyl residue on the outer acrosomal membrane glycoprotein; SYBR14, freely travel through living cells and combine with nucleic acid.

After sperm cells were incubated at 37°C for 40 min, the upper sperm was taken, and then, sperm performance parameters were determined according to the kit’s operating instructions. At pH of 6.2 and 7.4, the sperm acrosome and plasma membrane integrity rates were not significant different from those of the control group (*P* > 0.05). Results are shown in Figure 3, and the specific values are shown in Table 2.

**TABLE 2:** Detection of sperm indices after incubating 40 min at different pH levels.

**Sperm quality**	**pH 6.8**	**pH 6.2**	**pH 7.4**
Acrosome intact rate (%)	92.15 ± 2.42	92.34 ± 2.26	91.26 ± 2.54
Membrane integrity (%)	83.22 ± 2.27	82.34 ± 2.55	86.48 ± 2.31
ATP content (nmol/10^7^ sperm)	11.14 ± 0.23	10.82 ± 0.15	12.41 ± 0.13
DNA fragmentation (%)	4.75 ± 0.94	5.22 ± 1.12	5.64 ± 0.76
High mitochondrial activity (%)	97.62 ± 3.25	95.44 ± 3.74	97.58 ± 2.17
Sperm ROS content (MFI × 100 AU)	76.24 ± 1.25	72.66 ± 1.53	76.49 ± 1.31
Total sperm motility rate (%)	58.13 ± 2.02	61.37 ± 1.18	62.55 ± 1.74
Sperm forward movement rate (%)	51.37 ± 1.72	53.83 ± 1.54	55.18 ± 1.45
Sperm straight line speed (μm/s)	38.25 ± 1.67	39.18 ± 1.54	40.66 ± 1.72
Sperm curve speed (μm/s)	27.21 ± 1.39	28.88 ± 1.34	28.16 ± 1.13
Sperm average speed (μm/s)	28.63 ± 1.37	29.20 ± 1.45	31.76 ± 1.15

*Values with different superscripts (a and b) within each row are significantly different from each other (*P* < 0.05). Experiments were run six times.*

### Effect of Different Hydrogen Ion Concentration Diluents on the Quality of the Separated Upper Sperm Cells

#### Adenosine Triphosphate Content

**FIGURE 4:**
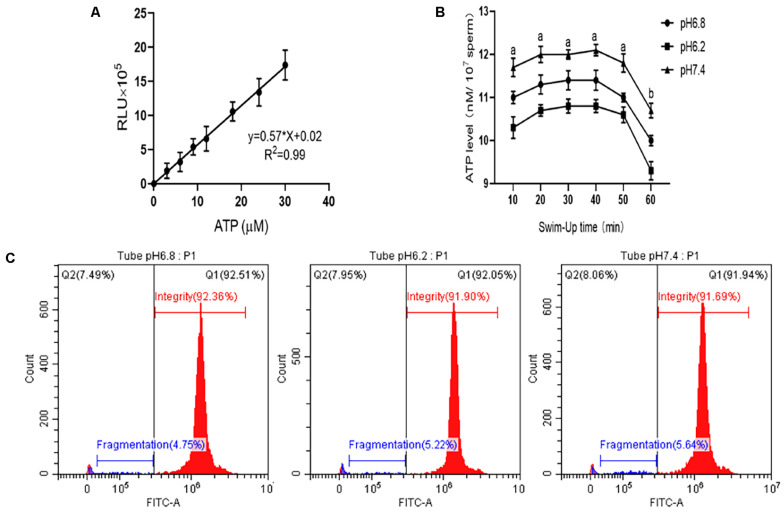
Detection of the ATP content and DNA integrity in the upper enriched sperm. **(A)** Standard curve of ATP content and fluorescence intensity in sperm cell samples. **(B)** ATP content changes in sperm cell samples at different incubation times. **(C)** DNA integrity test results when sperm cells were incubated in different pH dilutions for 40 min. “a,b” mean significantly different from each other (*P* < 0.05).

In this study, we established a standard curve with gradient dilutions of ATP standards and fluorescence intensity (*y* = 0.5734*x*, *R*^2^ = 0.9923), as shown in Figure 4A. Changes in the internal ATP content of sperm cells during the incubation process were then determined. The results are shown in Figure 4B. Within 60 min of incubation, no significant difference in the sperm’s internal ATP content was observed between the experimental and control groups at pH 6.2 and 7.4 (*P* > 0.05). However, all sperm ATP content decreased significantly after incubation for more than 50 min (*P* < 0.01).

#### DNA Integrity

When the sperm was incubated for 40 min, the upper sperm was taken, and the DNA integrity of the sperm was tested (Figure 4C). The sperm DNA integrity at pH 6.2 and 7.4 was not significant different from that of the control group (*P* > 0.05).

#### Mitochondrial Activity and Reactive Oxygen Species Content

**FIGURE 5:**
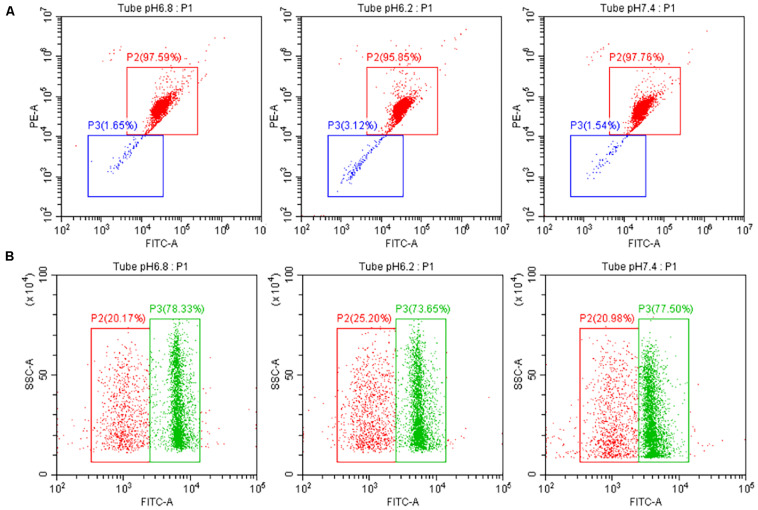
Test the quality of the upper enriched sperm cell samples. **(A)** Test result of the mitochondrial activity of separated sperm cells with different pH dilutions. The red and green boxes indicate high and low sperm mitochondrial activity, respectively. **(B)** Test result of the ROS content of separated sperm cells with different pH dilutions. The green and red boxes indicate high and low ROS content in sperm, respectively.

The results of the tested sperm cell sample’s mitochondrial membrane potential are shown in Figure 5A. No significant difference in the sperm’s mitochondrial activity was observed between the experimental and control groups at pH 6.2 and 7.4 (*P* > 0.05).

The results of the ROS detected in sperm cells are also shown in Figure 5B. When the pH values of the experimental group were 6.2 and 7.4, the level of ROS produced by sperm cell samples was not significant different from that of the control group (*P* > 0.05).

#### Motility Activity

**FIGURE 6:**
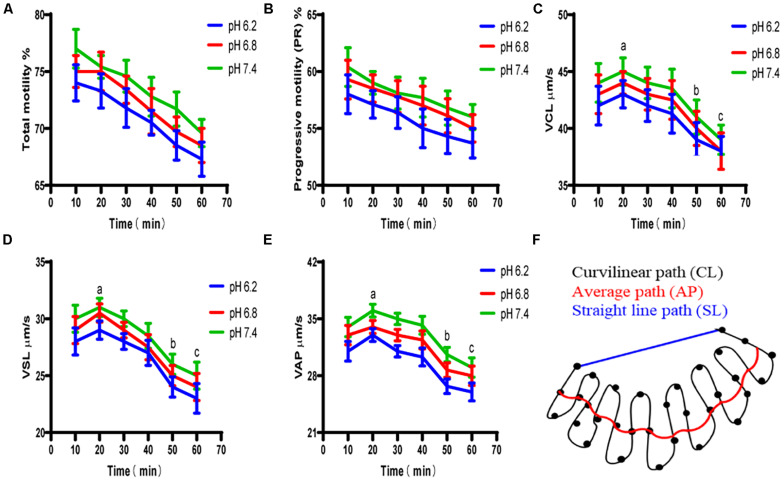
The motility activity of the upper enriched sperm cells at different pH dilutions. **(A)** Proportion of total motile sperm. **(B)** Proportion of sperm that moved forward. **(C)** Sperm cell’s curve velocity. **(D)** Sperm cell’s linear velocity. **(E)** Sperm cell’s average path velocity. **(F)** Average curve velocity, straight-line velocity, and schematic of the average path speed trajectory. “a,b,c” mean significantly different from each other (*P* < 0.05).

Test results for the upper enriched sperm cell’s motility activity are shown in Figure 6. When the pH values of the diluted solution in the experimental group were 6.2 and 7.4, the total sperm motility ratio and forward motility ratio of the experimental group were not significant different from those of the control group (*P* > 0.05). The specific values of the sperm quality tested above are shown in Table 2.

### Sex Ratio of Embryos for *in vitro* Fertilization

**FIGURE 7:**
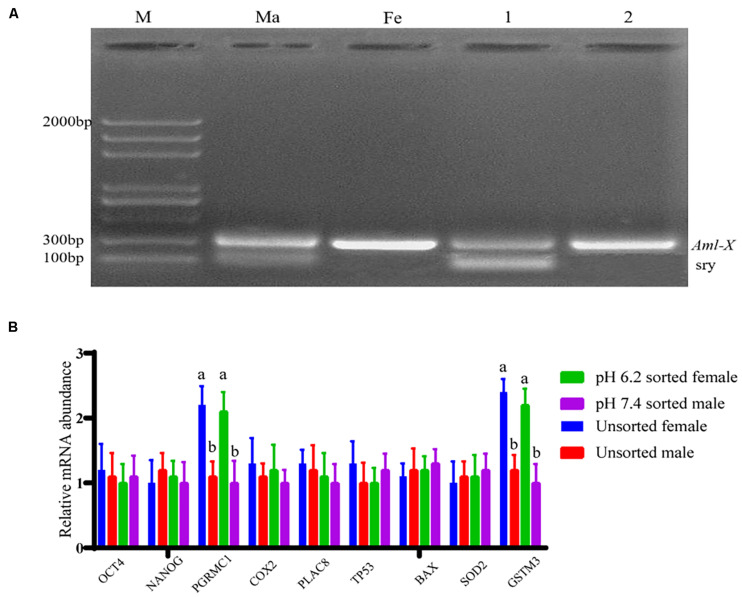
Partial embryo gender identification and blastocyst quality related gene detection. **(A)** The duplex PCR amplification product gel electrophoresis results. Lane M is 2,000 bp DNA ladders; lane Ma is the male goat DNA; lane Fe is female goat DNA; lane 1 is male embryos; lane 2 is female embryos. **(B)** Relative mRNA expression of nine genes relating to blastocyst quality. “a,b” mean significantly different from each other (*P* < 0.05).

The fertilization rate was calculated based on the release of the second polar body and the formation of a zygote between the female pronucleus and the male pronucleus as the basis for successful fertilization. The duplex PCR method was then used to amplify the genomic DNA of each blastocyst to identify the sex of the embryo, part of the gel image is shown in Figure 7A. When the pH of the dilution solution was 6.2 and 7.4, the upper sperm collected for *in vitro* fertilization, and the fertilization rate and blastocyst development rate were not significantly different from those of the control group (*P* > 0.05). The proportion of female embryos was 66.67% ± 0.05% and 29.73% ± 0.04%, respectively, which is significantly different from the control group 48.57% ± 0.02% (*P* < 0.01). Results are shown in Table 3.

**TABLE 3:** Sperm *in vitro* fertilization rates and embryo sex ratios.

**Diluent pH**	**Upper sperm X ratio (%)**	**Fertilization rate (%)**	**Blastocyst rate (%)**	**Female embryo (%)**
pH 6.8	52.35 ± 1.72^b^	60.00 ± 5.78 (54/90)^a^	38.89 ± 3.85 (35/90)^a^	48.57 ± 0.02 (17/35)^b^
pH 6.2	67.24 ± 2.61^a^	57.78 ± 1.93 (52/90)^a^	36.67 ± 3.33 (33/90)^a^	66.67 ± 0.05 (22/33)^a^
pH 7.4	30.45 ± 1.03^c^	63.33 ± 3.33 (57/90)^a^	41.11 ± 1.93 (37/90)^a^	29.73 ± 0.04 (11/37)^c^

*Values with different superscripts (a and b) within columns are significantly different from each other (*P* < 0.05). Experiments were run three times.*

### mRNA Expression of Genes Relating to the Developmental Potential of Blastocysts

The mRNA expression of genes relating to the developmental potential of blastocysts was examined using a relative qPCR method, and the results are shown in Figure 7B. No significant difference was observed in the gene expression between blastocysts of the same sex formed after fertilization with separated and unseparated sperm cell samples (*P* > 0.05). Nevertheless, the mRNA expression levels of PGRMC1 and GSTM3 were higher in female blastocysts formed after fertilization with isolated or unisolated sperm than in male blastocysts (*P* < 0.01).

## Discussion

Sex control technologies can interfere with the reproductive process of animals to select the desired sex offspring, which is of great importance for livestock production. Many economically important traits in livestock production are related to sex, such as meat, eggs, milk, hair, and velvet, which requires animals of a specific sex for production (Johnson, 2000; Dahlen et al., 2014). The easiest way to perform this separation is to split X and Y sperm cells before fertilization (Cran, 2007). By far, the most efficient and frequently used sperm separation method is flow cytometry (Soares et al., 2019), which is widely used in the pig and cattle industries and possesses significant benefits (Vazquez et al., 2009; Garner et al., 2013). Nevertheless, the X and Y sperm sorting system in dairy goats has not yet been marketed and scaled up. This scale-up proves difficult because no mature sorting parameters exist, the sorting speed is slow, and it cannot meet the requirements of large-scale sperm transfusion. Furthermore, the viability of sperm decreases after staining and sorting, and the equipment is expensive. It also requires specialized personnel to operate. Therefore, it is currently not widely used in dairy goat sperm sorting (Xie et al., 2020).

Recently, studies have been conducted to separate the X/Y spermatozoa by changing sperm incubation conditions. Madrid-Bury et al. (2003) isolated the X/Y spermatozoa from Holstein bulls using an upstream method (addition of heparin), and the percentage of Y spermatozoa in the upper sperm decreased after 150 min of incubation. Asma Ul et al. (2017) also isolated X/Y spermatozoa from buffaloes using a modified upstream method. He discovered that the percentage of Y sperm cells accounted for four times more than the X sperm (Asma Ul et al., 2017). In addition, the researchers studied the relationship between pH and the sex ratio of offspring by measuring the pH of the reproductive tract during insemination or changing the pH of the semen incubation solution. Wakim (1972) reported that the pH of the cervix of rabbits was positively correlated with the proportion of male offspring. Muehleis and Long (1976) reported that only changing the pH of the dilution had no significant effect on the sex ratio of offspring. Pratt et al. (1987) found that when Mesocricetus auratus mate in mid-estrus, there was a significant negative correlation between vaginal pH and the sex ratio of subsequent litters. Oyeyipo et al. (2017) incubated human spermatozoa at different pH dilutions and found that the proportion of the X and Y spermatozoa at the upper sperm increased by 12.7 and 8.9% in acidic and alkaline pH environments, respectively. However, some studies have found that changing the pH of the diluent does not separate X/Y spermatozoa. Raval et al. (2019) separated X/Y spermatozoa in buffaloes by changing the incubation fluid pH. Results from their study showed no significant difference in the X/Y spermatozoa in the upper sperm (Raval et al., 2019). In this study, the double TaqMan qPCR method was used to calculate the X/Y sperm ratio of dairy goat sperm after incubation at different pH dilutions for different times. Results showed that the percentage of the X sperm cells in the upper sperm was 67.24% ± 2.61% when the sperm dilution pH was 6.2 and incubated at 37°C for 40 min, which was significantly higher than that of the control group 52.35% ± 1.72%. However, the percentage of Y sperm cells in the upper sperm was 69.53% ± 3.04% when the dilution pH was 7.4 and incubated at 37°C for 40 min, which was significantly higher than that of the control group 48.12% ± 2.18%. Therefore, these results proved that the X/Y sperm of dairy goats can be effectively enriched using different pH of the diluent. Interestingly, we found that when the pH of the diluent was lower than 6.2 (pH 5.8) and higher than 7.4 (pH 7.8), the proportions of X sperm and Y sperm decreased. Based on the analysis of Zhou et al.’s research results, it may be related to the pH tolerance range of sperm (Zhou et al., 2015).

The environmental pH of spermatozoa is critical for their biological activity. Studies have shown that intracellular pH in mammalian spermatozoa is regulated by a combination of Hv1 channels (proton-gated channels), the HCO_3_^–^ system, and sodium–hydrogen ion-exchange channels (NHE) (Chauhan et al., 2017; Mishra et al., 2019). Furthermore, pH changes have effects on enzymes, hormones, transmitters, and growth factors in spermatozoa (Nishigaki et al., 2014; Mishra et al., 2018). Sperm intracellular pH also regulates specific calcium channel activity in the flagellum (CatSper), which is further involved in processes such as sperm hyperactivation, energization, and acrosome reaction (Darszon et al., 2005; Darszon et al., 2011). Additionally, intracellular pH is directly related to sperm viability, with higher values of sperm kinetic parameters, membrane integrity, and mitochondrial activity at pH 7 and 7.5 being observed (Jones and Bavister, 2000; Rizvi et al., 2009; Contri et al., 2013). The external pH of the sperm directly influences the pH of the sperm cell’s cytoplasm, which is linearly related to the extracellular pH (Mishra et al., 2018). In this study, a linear relationship was found between the internal and external sperm pH concentrations, *y* = 0.56*x* + 2.78, *R*^2^ = 0.98, thereby providing a convenient way to study the relationship between the internal pH and functional parameters of spermatozoa in the future. Sperm cells under different pH dilutions have differences in motor activity, DNA damage degree, and expression of apoptotic protein (You et al., 2017), including membrane surface ion channel (Ca^2+^, k^+^, Na^+^, etc.) activity (Miller et al., 2015; Nowicka-Bauer and Szymczak-Cendlak, 2021) and sperm tail VSP protein. The level of phosphorylation varies (Zhelay et al., 2018). Zhou et al. (2015) found that total sperm motility and progressive motility were higher at pH 7.2 and 8.2 than at pH 5.2 and 6.2. They also found that ATPase activity decreased and sperm acquisition was reduced when sperm cells were subjected to acidic pH (Zhou et al., 2015). You et al. (2017) studied the effect of pH on X/Y sperm motility, degree of DNA damage, and apoptotic protein expression. They found that Y sperm cells were more susceptible to environmental changes, with reduced motility and higher degree of DNA damage than X sperm cells (You et al., 2017). In this study, the motility activity of spermatozoa incubated under different pH dilutions at different times was analyzed using computer-assisted sperm analysis (CASA). The highest and lowest sperm motility indices were found at pH 7.4 and 6.2, respectively. It indicates that sperm motility activity increases with an increase in pH.

Studies have reported that the X/Y spermatozoa separated for *in vitro* fertilization suffer from low fertilization rates mainly due to the damage to the spermatozoa during sorting, including dilution, centrifugation, incubation, exposure to DNA stains, high pressure, lasers, and electrical charges (Bermejo-Alvarez et al., 2008). Changes in motor activity (Suh et al., 2005), shorter survival time (Hollinshead et al., 2003), accelerated acrosome reaction (Mocé et al., 2006), and increased sperm capacitation in advance (Maxwell et al., 1998) are further damages to sperm cells. The sperm acrosome contains many hydrolytic enzymes that are closely related to fertilization, and acrosome integrity facilitates the fertilization process (van der Horst, 2021). Several mitochondria are distributed at the neck of the sperm, which provides energy for sperm metabolism and movement, and participate in several fertilization processes, such as sperm hyperactivation, capacitation, and acrosome reaction (Nesci et al., 2020; Raad et al., 2021). Additionally, ROS is the main free radical in sperm cells. Therefore, normal physiological levels of ROS regulate sperm maturation, energetic acquisition, activation, acrosome reaction, and sperm–egg fusion processes, whereas excess ROS causes sperm pathological responses, such as free radical-induced lipid peroxidation (LPO), DNA damage, and apoptotic phenomena (Aitken, 2017; Zhang et al., 2019). In this study, the performance of sperm separated by the dilution pH of 6.2 and 7.4 was tested. It was found that the acrosome and plasma membrane intact rates were above 90 and 80%, respectively, which would not affect *in vitro* fertilization. The results of sperm quality test also found that DNA fragmentation of separated sperm was less than 10%, and the ATP content was higher than 10 nmol/10^7^ sperm. The proportion of high mitochondrial activity sperm was higher than 90%, whereas the ROS content and various motility activities were no different from those of the control group. Results of the *in vitro* fertilization performed using the isolated spermatozoa also showed that the fertilization rates were 57.78 ± 1.93 and 63.33 ± 3.33% at diluent pH of 6.2 and 7.4, respectively, which were not significantly different from those of the control group 60.00 ± 5.78%. Furthermore, the percentages of development to blastocyst were 36.67 ± 3.33 and 41.11 ± 1.93%, respectively, which were not significantly different from the control group (38.89 ± 3.85%), proving that the isolation method had no effect on sperm performance and quality. The sex of *in vitro* fertilized embryos was detected using the duplex PCR method, and the percentages of female offspring were 66.67 ± 0.05 and 29.73 ± 0.04% at dilution pH 6.2 and 7.4, respectively. This result was similar to the percentages obtained from X sperm cell samples in the isolated upper sperm and highly significantly different from that of the control group at 48.57% ± 0.02%.

DNA damage occurs in sperm cells during isolation, which affects blastocyst development (Blondin et al., 2009; Casanovas et al., 2019). In this study, the mRNA expression of TP53 (Paskulin et al., 2012) and BAX (Majidi Gharenaz et al., 2018), which is related to pro-apoptosis, was detected, but no difference between isolated and non-isolated spermatozoa was observed. Studies have reported abnormalities in gene expression or ultrastructure of embryos produced by sorted sperm cells (Gutiérrez-Adán et al., 2000; Morton et al., 2007; Palma et al., 2008), and differences in embryo metabolism (Kimura et al., 2005) and epigenetic status (Bermejo-Alvarez et al., 2008) have been highlighted. OCT4 and NANOG genes contribute to maintaining the pluripotency and self-renewal of embryonic stem cells (Mitsui et al., 2003; Stirparo et al., 2021). PGRMC1, which is a membrane-bound progesterone receptor that mediates the antiapoptotic effects of progesterone in granulosa cells, has also been discovered (Peluso et al., 2006; Cho et al., 2020). Furthermore, prostaglandin G/H synthase 2 (COX2),which is related to prostaglandin synthesis and is more abundant in blastocysts result in calf delivery compared with those resulting in resorption (Sen Roy and Seshagiri, 2013). However, PLAC8 is an invasion-specific gene that is more abundant in blastocysts, resulting in calf delivery compared with those resulting in resorption (Stamperna et al., 2020). Additionally, GSTM3 is involved in detoxifying electrophilic compounds, such as oxygen radicals, by conjugation with glutathione (Bermejo-Alvarez et al., 2010), whereas superoxide dismutase 2 (SOD2) is an important endogenous antioxidant enzyme in the body (Marques et al., 2018). In this study, we found no difference in gene expression between the above genes in blastocysts formed after fertilization of isolated and non-isolated sperm cells. Nevertheless, the mRNA expression of PGRMC1 and GSTM3 was significantly higher in female blastocysts than in male blastocysts, both in isolated and non-isolated spermatozoa, which is proposed to be related to the metabolic, antioxidation (Gutiérrez-Adán et al., 2000; Chanas et al., 2002), and antiapoptotic (Peluso et al., 2006) reactions between male and female blastocysts. It has been reported that DNA hypermethylation regulates the expression of GSTM3, which provides a possible link between activation of the glutathione pathway and epigenetic status because a higher methylation level has been reported in male blastocysts (Bermejo-Alvarez et al., 2008). In conclusion, *in vitro* fertilization using diluent pH isolated sperm has no effect on blastocyst development potential.

## Conclusion

In this study, when the pH of the sperm diluent is 6.2 and 7.4, X sperm and Y sperm can be enriched respectively, and no significant effect was observed on the sperm cell’s performance and quality. Using enriched sperm and mature oocytes from dairy goats for *in vitro* fertilization, female embryos accounted for 66.67% ± 0.05 and 29.73% ± 0.04%, respectively. Results show that this study has successfully established a simple and effective method to achieve gender control of dairy goats using the sperm pH dilution method.

## Data Availability Statement

The original contributions presented in the study are included in the article/Supplementary Material, further inquiries can be directed to the corresponding authors.

## Ethics Statement

The animal study was reviewed and approved by the Animal Ethics Committee of Northwest A&F University approved the experimental procedures. All experimental procedures were conducted in accordance with the Guide for the Care and Use of Laboratory Animals (Ministry of Science and Technology of China, 2006).

## Author Contributions

QH performed most experiments with assistance from SW, MH, YW and KZ. QH, JK, YZ, and FQ designed the experiments and analyzed the results. QH and FQ wrote the manuscript. All authors read, corrected, and approved the final manuscript.

## Conflict of Interest

The authors declare that the research was conducted in the absence of any commercial or financial relationships that could be construed as a potential conflict of interest.

## Publisher’s Note

All claims expressed in this article are solely those of the authors and do not necessarily represent those of their affiliated organizations, or those of the publisher, the editors and the reviewers. Any product that may be evaluated in this article, or claim that may be made by its manufacturer, is not guaranteed or endorsed by the publisher.

## Abbreviations

pH: hydrogen ion concentration
pHi: sperm cell internal pH
pHo: sperm cell external pH
EDTA: ethylene diamine tetraacetic acid
CASA: computer-assisted sperm analysis
COC: cumulus–oocyte complexes
MFI: mean fluorescence intensity
qPCR: real-time quantitative polymerase chain reaction
BCECF-AM: 2′, 7′ -bis-(2-carboxyethyl)-5(6)-carboxyfluorescein, acetoxymethyl ester
ROS: reactive oxygen species
PNA: peptide nucleic acid
ATP: adenosine triphosphate.
