# Supplementary material for: Effects of Diluent pH on Enrichment and Performance of Dairy Goat X/Y Sperm
Source: Front Cell Dev Biol. 2021 Oct 1;9:747722. doi: 10.3389/fcell.2021.747722 (PMC8517142; doi:10.3389/fcell.2021.747722)
Supplement: Supplementary file 1 [file Data_Sheet_1.docx]

Supplementary Material

Operating instructions for the kit used to determine the performance and quality of sperm.

### Operating instructions of the sperm acrosome integrity test kit:

1. Measure 10 μL of upper semen after incubation, calculate the density with a blood cell counting plate, and adjust the density to 200 × 10^4^/mL.

2. Measure 100 μL of semen, add 10μL of Hoechst33258 fluorescent dye, and incubate for 5 min at room temperature in the dark.

3. Add 500 μL of cleaning solution and mix well, centrifuge at 600 × g for 5min, and discard the supernatant.

4. Add 200 μL of PNA-FITC fluorescent dye, and incubate for 20 min at 25 ± 2 ℃ in the dark.

5. Add 500 μL of cleaning solution and mix well, centrifuge at 600 × g for 5 min, and discard the supernatant.

6. Repeat step 6 and add 1mL diluent for flow cytometry analysis.

### Operating instructions of the sperm plasma membrane integrity test kit:

1. Measure 10 μL of upper semen after incubation, calculate the density with a blood cell counting plate, and adjust the density to 200 × 10^4^/mL.

2. Measure 100 μL of semen, add 10 μL of PI fluorescent dye, and incubate for 10 min at 25 ± 2 ℃ in the dark.

3. Add 500 μL of cleaning solution and mix well, centrifuge at 600 × g for 5min, and discard the supernatant.

4. Add 5 μL of SYBR14 fluorescent dye, and incubate for 10min at 25 ± 2 ℃ in the dark.

5. Add 500 μL of cleaning solution and mix well, centrifuge at 600 × g for 5min, and discard the supernatant.

6. Repeat step 6 and add 1 mL diluent for flow cytometry analysis.

### Instructions for sperm DNA fragmentation detection:

1. Measure 10 μL of upper semen after incubation, calculate the density with a blood cell counting plate, and adjust the density to 200 × 10^4^/mL.

2. Measure 100 μL of semen, add 10 μL of acridine orange (AO) fluorescent dye, and incubate for 20 min at 25 ± 2 ℃ in the dark.

3. Add 500 μL of cleaning solution and mix well, centrifuge at 600× g for 5min, and discard the supernatant.

4. Repeat step 4, add 1mL diluent for flow cytometry analysis.

### Instructions for the detection of sperm ATP content:

1. Preparation of ATP standard curve: Dilute the 3 mmol/L ATP standard solution to 3 μmol/L, 6 μmol/L, 9 μmol/L, 12 μmol/L, 18 μmol/L, 24 μmol/L and Different gradient concentration standard solutions of 30 μmol/L.

2. Preparation of ATP detection working solution: Prepare the working solution at the ratio of 95 μL Assaybuffer + 1 μL subatrate + 1 μL ATP Enzyme, and store on ice.

3. Measure 10 μL of upper semen after incubation, calculate the density with a blood cell counting plate, and adjust the density to 200 × 10^4^/mL.

4. Measure 100 μL of semen, add 200 μL of lysis buffer to lyse, and place on ice for lysis for 15 min.

5. Add 90 μL of detection working solution, and use a multifunctional microplate reader to quickly detect the fluorescence intensity.

### Instructions for sperm mitochondrial activity detection kit:

1. Measure 10 μL of upper semen after incubation, calculate the density with a blood cell counting plate, and adjust the density to 200 × 10^4^/mL.

2. Measure 100 μL of semen, add 10 μL of JC-1 fluorescent dye, and incubate for 20 min at 25 ± 2 ℃ in the dark.

3. Add 500 μL of cleaning solution and mix well, centrifuge at 600 × g for 5min, and discard the supernatant.

4. Repeat step 4, add 1mL diluent for flow cytometry analysis.

### Instructions for sperm ROS content detection kit:

1. Prepare the fluorescent probe DCFH-DA working solution (5μmol/L).

2. Measure 10 μL of upper semen after incubation, calculate the density with a blood cell counting plate, and adjust the density to 200 × 10^4^/mL.

3. Measure 100 μL of semen, add 10 μL of DCFH-DA working solution, and incubate for 10 minutes at 25 ± 2 ℃ in the dark.

4. Add 500 μL of cleaning solution and mix well, centrifuge at 600 × g for 5min, discard the supernatant.

5. Repeat step 5, add 1mL diluent for flow cytometry analysis.

### Instructions for detecting sperm motility index:

1. Measure 10 μL of upper semen after incubation, calculate the density with a blood cell counting plate, and adjust the density to 200 × 10^4^/mL.

2. Measure 10 μL of semen and drop it on the preheated glass slide.

3. Place the slide on the 37°C constant temperature heating plate to capture the sperm trajectory.

4. Randomly select 5 fields for analysis, and analyze the sperm movement through software.
